# Clinical course of untreated cerebral cavernous malformations: a meta-analysis of individual patient data

**DOI:** 10.1016/S1474-4422(15)00303-8

**Published:** 2016-02

**Authors:** Margaret A Horne, Kelly D Flemming, I-Chang Su, Christian Stapf, Jin Pyeong Jeon, Da Li, Susanne S Maxwell, Philip White, Teresa J Christianson, Ronit Agid, Won-Sang Cho, Chang Wan Oh, Zhen Wu, Jun-Ting Zhang, Jeong Eun Kim, Karel ter Brugge, Robert Willinsky, Robert D Brown, Gordon D Murray, Rustam Al-Shahi Salman, Cerebral Cavernous Malformations Individual Patient Data Meta-analysis Collaborators

**Affiliations:** a Centre for Population Health Sciences, University of Edinburgh, Edinburgh, UK; b Centre for Clinical Brain Sciences, University of Edinburgh, Edinburgh, UK; c Department of Neurology, Mayo Clinic, Rochester, MN, USA; d Division of Biomedical Statistics and Informatics, Mayo Clinic, Rochester, MN, USA; e Division of Neuroradiology, Department of Medical Imaging, Toronto Western Hospital, Toronto, ON, Canada; f Département Hospitalo-Universitaire (DHU) NeuroVasc, Hôpital Lariboisière, Paris, France; g Department of Neurosurgery, Hallym University College of Medicine, Chuncheon, South Korea; h Department of Neurosurgery, Beijing Tiantan Hospital, Capital Medical University, Beijing, China; i Institute of Neuroscience, Newcastle University, Newcastle-upon-Tyne, UK; j Department of Neurosurgery, Seoul National University College of Medicine, Seoul, South Korea

## Abstract

**Background:**

Cerebral cavernous malformations (CCMs) can cause symptomatic intracranial haemorrhage (ICH), but the estimated risks are imprecise and predictors remain uncertain. We aimed to obtain precise estimates and predictors of the risk of ICH during untreated follow-up in an individual patient data meta-analysis.

**Methods:**

We invited investigators of published cohorts of people aged at least 16 years, identified by a systematic review of Ovid MEDLINE and Embase from inception to April 30, 2015, to provide individual patient data on clinical course from CCM diagnosis until first CCM treatment or last available follow-up. We used survival analysis to estimate the 5-year risk of symptomatic ICH due to CCMs (primary outcome), multivariable Cox regression to identify baseline predictors of outcome, and random-effects models to pool estimates in a meta-analysis.

**Findings:**

Among 1620 people in seven cohorts from six studies, 204 experienced ICH during 5197 person-years of follow-up (Kaplan-Meier estimated 5-year risk 15·8%, 95% CI 13·7–17·9). The primary outcome of ICH within 5 years of CCM diagnosis was associated with clinical presentation with ICH or new focal neurological deficit (FND) without brain imaging evidence of recent haemorrhage versus other modes of presentation (hazard ratio 5·6, 95% CI 3·2–9·7) and with brainstem CCM location versus other locations (4·4, 2·3–8·6), but age, sex, and CCM multiplicity did not add independent prognostic information. The 5-year estimated risk of ICH during untreated follow-up was 3·8% (95% CI 2·1–5·5) for 718 people with non-brainstem CCM presenting without ICH or FND, 8·0% (0·1–15·9) for 80 people with brainstem CCM presenting without ICH or FND, 18·4% (13·3–23·5) for 327 people with non-brainstem CCM presenting with ICH or FND, and 30·8% (26·3–35·2) for 495 people with brainstem CCM presenting with ICH or FND.

**Interpretation:**

Mode of clinical presentation and CCM location are independently associated with ICH within 5 years of CCM diagnosis. These findings can inform decisions about CCM treatment.

**Funding:**

UK Medical Research Council, Chief Scientist Office of the Scottish Government, and UK Stroke Association.

## Introduction

Cerebral cavernous malformations (CCMs) are the second commonest incidental vascular finding—after aneurysms^1^—on brain MRI, with a prevalence of one in 625 neurologically asymptomatic people.^2^, ^3^ Because brain MRI is needed for diagnosis of CCMs without pathological examination,^4^ the number of people in whom CCM has been detected has risen since the advent of MRI.^5^, ^6^

CCMs can be asymptomatic or can cause epileptic seizures,^7^ stroke due to symptomatic intracranial haemorrhage (ICH),^8^ or new focal neurological deficit (FND) without evidence on brain imaging of recent haemorrhage.^8^ As MRI use increases over time,^9^ so too does the need for information about the magnitude and predictors of the risk of ICH or FND from CCMs. These risks can inform decisions about whether to treat CCMs with neurosurgical excision or stereotactic radiosurgery, although the use of the latter remains controversial and neither treatment has been assessed in a randomised trial.^10^

Some cohort studies have estimated the risk of ICH from untreated CCMs. However, findings from a recent systematic review^11^ showed that these studies were mostly retrospective hospital-based series, with sample sizes not exceeding 139 people and short durations of follow-up, without clearly defined diagnostic criteria or outcome events,^8^ and in which several different statistical methods were used to calculate the risk and predictors of ICH. Findings from these studies have left uncertainty about the magnitude of the risk of ICH and its predictors. Additionally, there remains an absence of prediction models.

Therefore, we sought to address these uncertainties by undertaking a systematic review and meta-analysis of individual patient data from cohort studies of people with CCMs with similar designs and outcome definitions.^12^, ^13^ By using consistent methods of analysis, we aimed to estimate the risks of first ICH or FND during follow-up and to identify predictors of these outcomes.

## Methods

### Study design

We undertook this study according to a protocol finalised on May 9, 2012, which was approved by the Cerebral Cavernous Malformations Individual Patient Data Meta-analysis Collaborators. Two authors with training in undertaking systematic reviews (MAH and RA-SS) used electronic search strategies (appendix p 2)^11^ to search Ovid MEDLINE and Embase from inception until April 30, 2015, for published cohort studies; they screened the bibliographies of studies for other potentially eligible cohorts, established their eligibility, and resolved any disagreements by discussion. Cohorts were eligible for inclusion regardless of language of publication if they included people aged 16 years or older—a common age cutoff for transition to adult services^14^—at the time of a definite diagnosis of CCM confirmed by brain MRI, and if they included at least symptomatic ICH due to CCM and death as outcomes after an inception point of first diagnosis of CCM but before first CCM treatment with neurosurgical excision or stereotactic radiosurgery. After sending a copy of the protocol and invitation to collaborate to the corresponding authors of the reports that described cohorts that were eligible for inclusion, followed by one reminder, we included cohorts from studies for which the study investigators confirmed their eligibility and provided patient-level data on baseline covariates, outcomes, and CCM treatment. We were unable to use aggregate data from cohorts for which individual patient data were unavailable because time-to-event analyses require patient-level data.

Research ethics committees or other entities overseeing the use of patients' data approved the collaborating cohorts. Cohorts shared only anonymised data, so neither individual consent nor specific approval for this individual patient data meta-analysis were required.^15^

### Data collection

Collaborating cohorts provided patient-level data at baseline (sex, mode of symptomatic presentation leading to diagnosis of CCMs, age at CCM diagnosis, date of CCM diagnosis, CCM multiplicity, and primary CCM location) and during follow-up (all outcome types and dates, all treatment types and dates, and date of last follow-up) for time-to-event analyses. We excluded people who had been first diagnosed when younger than 16 years or who had already received treatment for CCMs.

Investigators in each cohort distinguished two clinical events attributable to CCMs, at presentation and during follow-up, where possible, according to the Angioma Alliance definitions.^8^ ICH was defined as acute or subacute onset of symptoms of haemorrhage with recent extralesional or intralesional haemorrhage confirmed by investigations (CT or MRI, or pathological examination at autopsy). FND was defined as new or worsened neurological deficit referable to the CCM anatomical location with or without timely investigation to rule out evidence of recent haemorrhage.^8^ We checked data from each cohort for internal consistency against published reports of the cohort and resolved any queries with the relevant collaborators. For missing data, we contacted collaborators to request the missing values. We assessed the risk of bias of each cohort according to an eight-item instrument published by the Cochrane Methods Bias group.^16^

### Outcomes

The primary outcome was first symptomatic ICH due to CCM. The secondary outcome was a composite of first symptomatic ICH or FND due to CCM. Time-to-event analyses started at diagnosis of CCM and terminated at the earliest occurrence of ICH only for the analysis of the primary outcome. For the secondary outcome, these analyses were terminated at the earliest occurrence of ICH or FND. If an outcome did not occur, we censored analyses at the earliest occurrence of CCM treatment, death unrelated to CCM, last available follow-up, or 5 years after CCM diagnosis.

### Statistical analysis

We agreed a detailed statistical analysis plan with collaborators in Oct 25, 2013, before data analysis began. We categorised mode of presentation as one of four mutually exclusive categories: ICH, FND, epileptic seizure if the seizure was neither symptomatic of a concomitant ICH nor more likely to be due to another cause, or incidental if a person was asymptomatic or if their symptoms (eg, headache) could not be ascribed to the underlying CCM. We attributed one CCM location to people harbouring more than one CCM by using the location of the symptomatic CCM; when a person presented asymptomatically with more than one CCM, brainstem CCM location took precedence since this location seemed to be a predictor of ICH from a systematic review of aggregate data from existing studies.^11^ We calculated the relative risk of having a CCM located in the brainstem for ICH or FND versus other presentations.

We used survival analysis to estimate the 5-year risk of symptomatic ICH attributable to CCMs. The inception point was the earliest date of definite diagnosis of CCMs by radiographic or pathological investigation.

We prespecified five potential predictors for investigation of their association with outcome on the basis of their clinical relevance, likelihood of being associated with outcome,^11^, ^17^, ^18^, ^19^, ^20^ reliability, accuracy of measurement, completeness, and availability at the time of diagnosis.^21^ Core predictors were mode of presentation (ICH or FND *vs* other) and CCM location (brainstem *vs* other). We dichotomised mode of clinical presentation because previous ICH seemed to be a predictor from a systematic review of aggregate data from existing studies, and included FND because in certain circumstances FND can suggest undetected ICH.^8^ Putative predictors were sex (female *vs* male), CCM multiplicity (more than one *vs* one), and increasing age at diagnosis (which we treated as a continuous variable in association analyses).^22^ We did separate univariable analyses of each cohort and the pooled data. We used Cox regression to calculate the unadjusted hazard ratios (HRs) for each predictor. We used log-minus-log plots to check that the proportional-hazards assumption was met before undertaking Cox proportional-hazards multivariable regression. In the statistical analysis plan, we specified that in multivariable adjusted analyses, we would enter the core predictors into the model first; then, to ascertain whether any of the remaining three putative predictors added significant information over and above the core predictors, we entered each putative predictor into the model, provided the conventional rule that there should be at least ten outcome events per predictor was fulfilled.^21^, ^23^ We followed this clinically driven approach because we were undertaking a two-stage individual patient data meta-analysis and we were aware that the number of events during follow-up in each individual cohort would be insufficient to permit a full data-driven selection of variables to be included in the model.

We did two-stage meta-analyses of the univariable associations of each of the five predictors with outcome, in which we derived study-specific unadjusted HRs and combined them using a random-effects model to generate a weighted unadjusted pooled HR.^24^, ^25^ We did meta-analyses for each of the three putative predictors, both unadjusted and adjusted for the core predictors. We assessed heterogeneity between studies using the *I*^2^ index of inconsistency, which measures the proportion of total variation in study estimates that is due to heterogeneity.^26^, ^27^

We planned to build prognostic models of the estimated 5-year risk of ICH after diagnosis on the basis of the findings of the multivariable analyses and meta-analyses.^23^, ^28^, ^29^ Because we had five potential predictors and outcome events tend to be infrequent, we used the entire dataset to develop our models, rather than split it into a derivation and a test set. For internal validation, we used bootstrapping to derive 95% CIs for the multivariable Cox regression analyses, by creating 10 000 random samples of the same size as the study cohort using sampling with replacement. We used Kaplan-Meier plots to assess the separation achieved by prognostic models, and life tables to estimate annual hazard rates of experiencing an ICH within 5 years of diagnosis of CCMs.

We did descriptive and survival analyses using IBM SPSS Statistics 19 and 21, and used Stata IC12 for the individual patient data meta-analysis.

### Role of the funding source

The funders of the study had no role in study design, data collection, data analysis, data interpretation, writing of the report, or in the decision to submit for publication. All authors had full access to all the data in the study, but only MAH and GDM analysed the data; the corresponding author had final responsibility for the decision to submit for publication.

## Results

**Figure 1:**
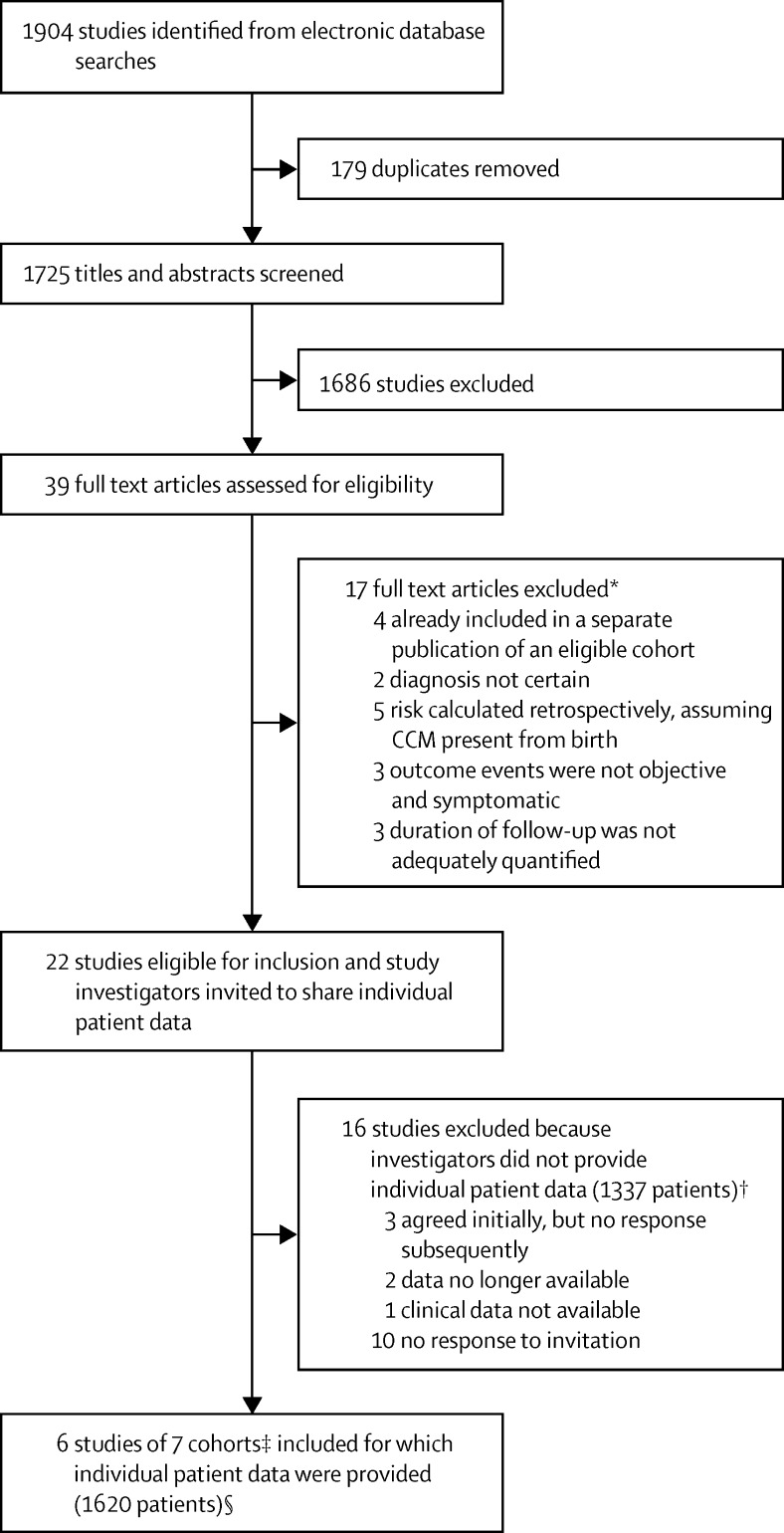
Study flowchart CCM=cerebral cavernous malformation. *See appendix pp 18–19. †See appendix pp 19–20 for references. ‡One eligible study provided data from two time periods, which are included as two separate cohorts. §See appendix p 20.

From 22 publications of potentially eligible cohorts that included 2957 people (figure 1; appendix p 3), six research groups provided published data on seven cohorts involving 1620 people for inclusion in this meta-analysis (appendix p 4).^11^, ^17^, ^18^, ^19^, ^20^, ^30^ For missing data, in every instance the investigators of the original study were able to check their database and provide the missing values for all covariates. Of the 16 research groups who did not join the collaboration, whose studies involved 1337 people (45% of the published data), three groups agreed to collaborate but did not share data, two groups no longer had access to their data, one group did not have clinical data available, and ten groups did not respond to our invitations (figure 1; appendix pp 19–20).

Five cohorts were from tertiary referral centres (Toronto Western Hospital, ON, Canada, 1987–2007, n=345; Mayo Clinic, Rochester, MN, USA, 1984–98, n=267; Hôpital Lariboisière, Paris, France, 1994–2011, n=81; Seoul National University Hospital, Seoul, South Korea, 1998–2010, n=326; and Beijing Tiantan Hospital, Beijing, China, 1985–2012, n=306) and one was from the Scottish population (1999–2003, n=135). Data from a seventh cohort (Scottish population, 2006–10, n=160) and 63% (217 of 345 adults) of the Toronto cohort were previously unpublished. The Beijing cohort was restricted to adults with a brainstem CCM. Follow-up was prospective in the population-based cohorts^11^ and one of the hospital-based cohorts,^20^ both retrospective and prospective in another hospital-based cohort,^17^ and retrospective in the remainder (appendix p 3).^18^, ^19^, ^30^ We did not identify problems (eg, incompatibility with different study inception points or differences in CCM, ICH, or FND definitions) when we checked the individual patient data. We used the date of symptom onset leading to diagnosis of CCM or the date of first medical assessment for 27 people without a date of diagnosis of CCMs in one cohort.^17^ All seven cohorts recorded FND as a mode of clinical presentation, but four cohorts did not record FND during follow-up.^18^, ^19^, ^20^, ^30^ Risk of bias in the seven cohorts was low (appendix p 5).

The median age at diagnosis was 45 years (range 16–91), 867 (54%) of people were women, 822 (51%) presented with ICH or FND, 282 (17%) had multiple CCMs, and 575 (35%) had CCMs located in the brainstem (table 1). People who had presented with ICH or FND were more likely to have a brainstem CCM than people who presented with a seizure or incidentally (prevalence ratio 6·0, 95% CI 4·8–7·5). The primary outcome event was recorded in all seven cohorts; follow-up ended at the occurrence of the first ICH outcome event (n=204) or censoring (owing to CCM treatment [n=193], death unrelated to ICH or FND [n=46], end of follow-up before 5 years [n=596], or end of follow-up at 5 years [n=581]). Total follow-up was 5197 person-years (median 3·5 years per person, IQR 1·6–5·0, 70% completeness^31^ of all potential follow-up). Only the Scottish and Toronto cohorts recorded both ICH and FND during follow-up (in 640 [40%] of 1620 included people), and so we restricted analysis of the secondary composite outcome event of ICH or FND to these cohorts.

**Figure 2:**
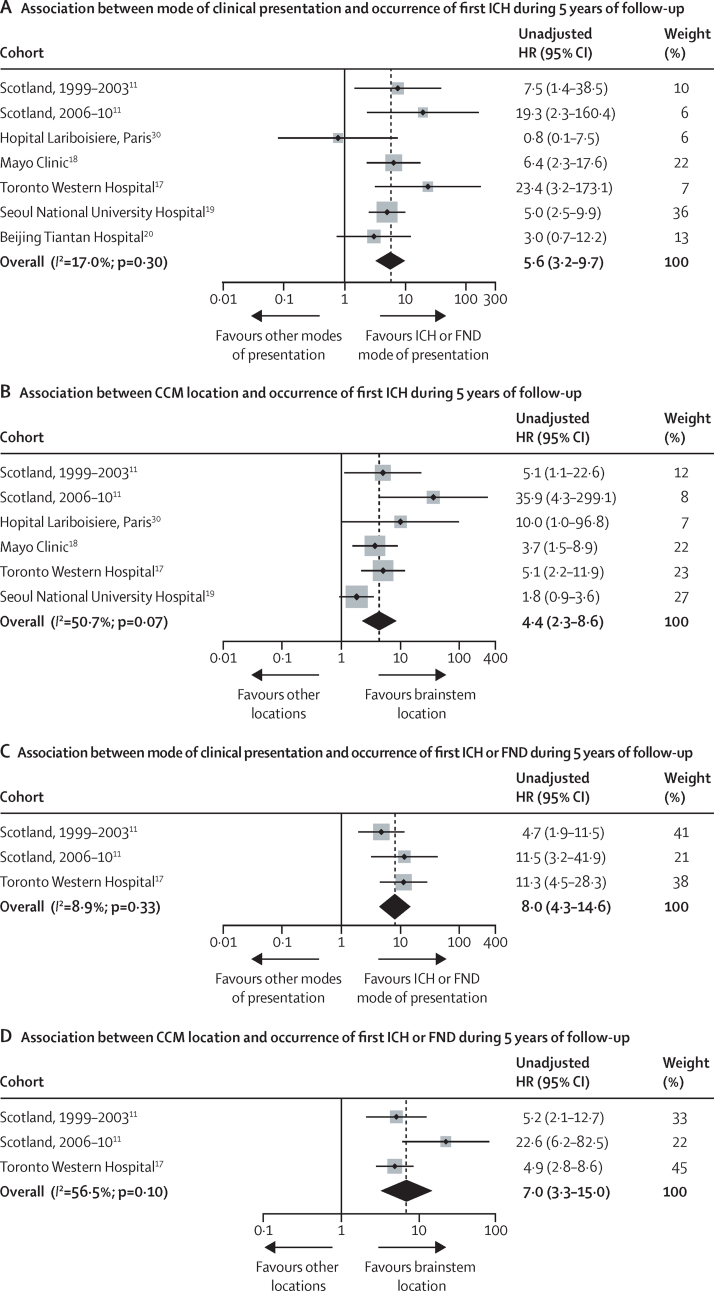
Forest plots of associations between mode of presentation and cerebral cavernous malformation location with primary and secondary outcomes Plots show cohort-level and pooled estimates of associations between ICH or FND at presentation (A and C) or brainstem CCM location (B and D) and outcome during 5 years of follow-up. The area of each shaded box is proportional to the weight of the cohort it represents. CCM=cerebral cavernous malformation. FND=focal neurological deficit. HR=hazard ratio. ICH=intracranial haemorrhage.

204 of 1620 people experienced ICH within 5 years of diagnosis of CCMs (Kaplan-Meier estimated 5-year risk 15·8%, 95% CI 13·7–17·9), four of which were fatal (1-month case fatality rate after ICH due to CCM; 2·0% [95% CI 0·1–3·9]). The estimated risk of first ICH within 5 years of diagnosis of CCM in the pooled dataset was higher for people presenting with ICH or FND versus other modes of presentation (26·4% [95% CI 23·1–29·7] *vs* 4·3% [2·5–6·1]; pooled unadjusted HR 5·6 [95% CI 3·2–9·7]) and for people with a primary CCM location in the brainstem versus another location (27·7% [95% CI 23·6–31·8] *vs* 8·2% [6·2–10·2]; pooled unadjusted HR 4·4, [95% CI 2·3–8·6]); these findings were similar in individual cohorts (figure 2; appendix pp 6–7). We found no evidence—even after multivariable adjustment for the two core predictors—that age, sex, or CCM multiplicity affected the risk of ICH, and these findings were generally consistent between cohorts (appendix pp 8–10).

We found no evidence of publication bias in a funnel plot of the HR for the primary outcome by mode of presentation (appendix p 11). We assessed risk of publication bias by inspecting a funnel plot of the HR against the standard error of log (HR). As a sensitivity analysis, we repeated the meta-analyses of the five cohorts with proportionally fewer outcome events. Although the Chinese cohort, which consisted entirely of adults with brainstem CCMs, contributed 44% of ICHs during follow-up (90 of 204), a sensitivity analysis excluding the Chinese and South Korean cohorts—both of which contained proportionally more people with ICH during follow-up than the other five cohorts (South Korea 52 of 204)—produced similar results (data not shown).

**Figure 3:**
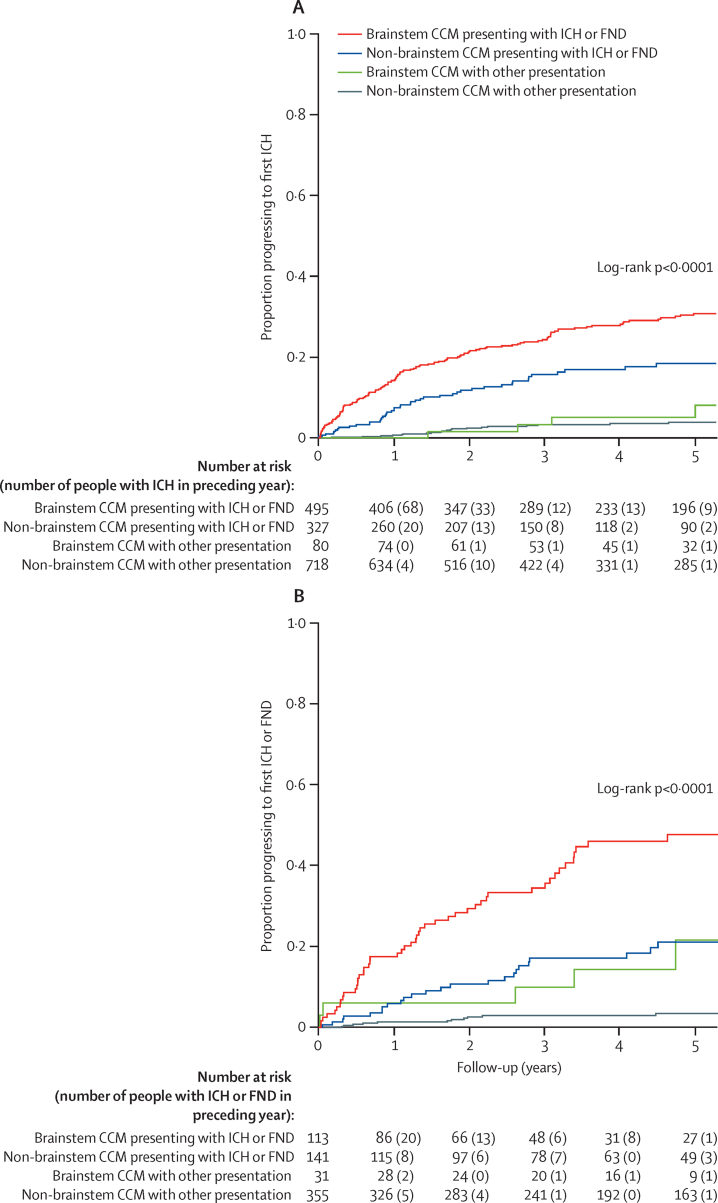
Kaplan-Meier plots of progression to intracranial haemorrhage or to intracranial haemorrhage or focal neurological deficit Plots show the proportion of people progressing to ICH (A) or ICH or FND (B) during follow-up, stratified by ICH or FND presentation from brainstem CCMs, ICH or FND presentation from non-brainstem CCMs, other presentation from brainstem CCMs, and other presentation from non-brainstem CCMs. CCM=cerebral cavernous malformation. FND=focal neurological deficit. HR=hazard ratio. ICH=intracranial haemorrhage.

The findings of the univariable and multivariable survival analyses led us to create two prognostic models, using the two core predictors—mode of clinical presentation and CCM location—in multivariable Cox regression analyses. By dichotomising each of these predictors, we identified four prognostic subgroups with significant differences in their risks of outcomes according to stratified Kaplan-Meier plots (figure 3; log-rank p<0·0001 for both proportion progressing to ICH and proportion progressing to ICH or FND) or according to Cox regression of 5-year event rates and HRs (table 2), after checking proportional hazards assumptions were met (appendix p 12). People with CCMs outside the brainstem who had not presented with ICH or FND were in the majority and had the lowest risk, whereas people with brainstem CCMs presenting with ICH or FND were the highest-risk group (figure 3; table 2; appendix p 13).

Estimates of the annual risk of ICH during each year of follow-up decreased from 6·2% (95% CI 4·9–7·4) in the first year of follow-up to 2·0% (0·9–3·0) in the fifth year overall (p<0·0001; appendix p 14). This reduction was evident only in people presenting with ICH or FND (appendix p 15).

In the subgroup of 640 people with data recorded for the composite secondary outcome of ICH or FND, 36 people had an ICH and 52 had an FND during follow-up. In this subgroup, the magnitude, direction, and consistency of associations of the core and putative predictors with the secondary outcome were similar to the primary analysis (table 2; figure 2; appendix pp 8–10 and 16–17), but the event rate was higher (17·0%, 95% CI 13·6–20·3).

## Discussion

To the best of our knowledge, this is the largest analysis of the clinical course of untreated CCMs so far, in which we found that brainstem CCM location and CCM presentation with ICH or FND were independently associated with the occurrence of ICH after diagnosis of CCMs, whereas age, sex, and CCM multiplicity did not contribute any additional prognostic information. The risk of ICH during 5 years of follow-up differed significantly according to the possession of either, or both, of these risk factors; annual ICH incidence decreased over time in people initially presenting with ICH or FND.

This study has the following strengths. The sample size of the pooled cohort was large and contained unpublished data in addition to data from people who were already described in published studies. There was 100% completeness for data on baseline covariates (appendix p 4). We ensured that outcome event definitions and statistical methods of survival analysis were consistent across the collaborating cohorts. The duration and completeness of follow-up enabled us to construct 5-year survival curves with more precise estimations of associations and event incidences than previously possible. The risk of bias in the participating cohorts was low, and the findings were consistent in retrospective hospital-based cohorts and prospective population-based cohorts (figure 2). These strengths in design are reinforced by robustness of the results when compared with findings from individual cohorts for which event rates of variable magnitude were estimated and for which findings of associations between outcome and our core and putative predictors have been inconsistent.^11^

This study has some limitations. Not all of the eligible published cohorts had retained data to share in this collaborative analysis. Outcome data were available on both ICH and FND for only 40% of the included people, but these data were sufficient to quantify event rates with reasonable precision and show associations between predictors and outcomes that were consistent with the primary analysis. Our statistical analysis assumed that censoring was non-informative, but even if informative censoring did occur (ie, because of treatment of CCMs that were in the brainstem or that presented with ICH or FND),^32^ it happened infrequently. We set a lower limit of 16 years for age at diagnosis, to focus this study on patients who were referred to neurologists and neurosurgeons who care for adults (ie, age at least 16 years), resulting in the exclusion of data on 46 (3%) of the 1666 people available in the collaborating cohorts; however, the proportion of events in people aged 16–18 years was comparable with the rest of the cohort (data not shown).

The main implication of our findings for clinical practice is that people with CCMs can be stratified into four groups to predict the 5-year risk of ICH. These risks can inform decisions about CCM treatment, by indirect comparison with estimates of the effects of treatment. Future research is needed to externally validate our prognostic model and establish whether other factors (eg, genotype, pregnancy, and statin or antithrombotic drug use) are independently associated with ICH in addition to the two core predictors; even larger sample sizes will be needed for this research. Furthermore, long-term risks remain to be quantified for a disorder that is often diagnosed in young people, but has only been well recognised by MRI since the 1980s.^4^

## Figures and Tables

**Table 1:** Baseline and follow-up characteristics

		**Mode of presentation leading to CCM diagnosis**				
		Incidental (n=461)	Seizure (n=337)	ICH (n=576)	FND (n=246)	Total (n=1620)
Age at diagnosis (years)		51 (37–62)	42 (30–57)	41 (32–51)	47 (36–60)	45 (33–58)
Sex						
	Female	259 (56%)	160 (47%)	310 (54%)	138 (56%)	867 (54%)
	Male	202 (44%)	177 (53%)	266 (46%)	108 (44%)	753 (46%)
More than one CCM		77 (17%)	70 (21%)	90 (16%)	45 (18%)	282 (17%)
Primary CCM location						
	Lobar	300 (65%)	289 (86%)	154 (27%)	69 (28%)	812 (50%)
	Deep	46 (10%)	18 (5%)	41 (7%)	24 (10%)	129 (8%)
	Cerebellum	50 (11%)	15 (4%)	22 (4%)	17 (7%)	104 (6%)
	Brainstem	65 (14%)	15 (4%)	359 (62%)	136 (55%)	575 (35%)
CCM management						
	Surgery or stereotactic radiosurgery	28 (6%)	77 (23%)	172 (30%)	35 (14%)	312 (19%)
	Conservative management	433 (94%)	260 (77%)	404 (70%)	211 (86%)	1308 (81%)
First outcome event during untreated follow-up*						
	ICH	12 (3%)	12 (4%)	151 (26%)	29 (12%)	204 (13%)
	FND	10 (2%)	3 (1%)	18 (3%)	24 (10%)	55 (3%)
	None	439 (95%)	322 (96%)	407 (71%)	193 (78%)	1361 (84%)
Censored follow-up (years)		3·9 (2·0–5·0)	3·6 (1·5–5·0)	3·0 (1·1–5·0)	4·2 (2·1–5·0)	3·5 (1·6–5·0)

Data are median (IQR) or number (%). Some percentages do not add up to 100 because of rounding. CCM=cerebral cavernous malformation. FND=non-haemorrhagic focal neurological deficit. ICH=intracranial haemorrhage.

* 1620 people contributed data on the occurrence of ICH outcomes. 640 people contributed data on the occurrence of the composite outcome of ICH or FND.

**Table 2:** Hazard ratios and estimated 5-year risks of outcome events for core predictors in prognostic models

	**Number of people (%)**	**Number of outcome events during 5-year follow-up**	Hazard ratio (95% CI)*	**Estimated 5-year risk (95% CI)**
**Primary outcome: ICH (n=1620)**				
ICH or FND presentation, brainstem CCM location	495 (31%)	135	10·2 (5·0–23·9)	30·8% (26·3–35·2)
ICH or FND presentation, other CCM location	327 (20%)	45	5·6 (3·7–9·4)	18·4% (13·3–23·5)
Other presentation, brainstem CCM location	80 (5%)	4	1·8 (1·3–2·6)	8·0% (0·1–15·9)
Other presentation, other CCM location	718 (44%)	20	Reference	3·8% (2·1–5·5)
**Secondary outcome: ICH or FND (n=640)**				
ICH or FND presentation, brainstem CCM location	113 (18%)	48	16·3 (5·8–53·7)	50·7% (40·1–61·4)
ICH or FND presentation, other CCM location	141 (22%)	24	5·1 (2·9–10·0)	22·4% (14·2–30·6)
Other presentation, brainstem CCM location	31 (5%)	5	3·2 (2·0–5·4)	22·9% (3·7–42·2)
Other presentation, other CCM location	355 (55%)	11	Reference	3·7% (1·5–5·9)

CCM=cerebral cavernous malformation. FND=non-haemorrhagic focal neurological deficit. ICH=intracranial haemorrhage.

* Bootstrapped 95% CIs: 10 000 samples with replacement.
