# Supplementary material for: Clinical course of untreated cerebral cavernous malformations: a meta-analysis of individual patient data
Source: Lancet Neurol. 2016 Feb;15(2):166–73. doi: 10.1016/S1474-4422(15)00303-8 (PMC4710581; doi:10.1016/S1474-4422(15)00303-8)
Supplement: Supplementary appendix [file mmc1.pdf]

THE LANCET Neurology

Supplementary webappendix

This webappendix formed part of the original submission and has been peer reviewed. We post it as supplied by the authors.

Supplement to: Horne MA, Flemming KD, Su I-C, et al, and the Cerebral Cavernous Malformations Individual Patient Data Meta-analysis Collaborators. Clinical course of untreated cerebral cavernous malformations: a meta-analysis of individual patient data. *Lancet Neurol* 2015; published online Dec 1. http://dx.doi.org/10.1016/S1474-4422(15)00303-8.

Appendix to:

Margaret A Horne, Kelly D Flemming, I-Chang Su, Christian Stapf, Jin Pyeong Jeon, Da Li, Susanne S Maxwell, Philip White, Teresa J Christianson, Ronit Agid, Won-Sang Cho, Chang Wan Oh, Zhen Wu, Jun-Ting Zhang, Jeong Eun Kim, Karel ter Brugge, Robert Willinsky, Robert D Brown Jr, Gordon D Murray, Rustam Al-Shahi Salman, and the Cerebral Cavernous Malformations Individual Patient Data Meta-analysis Collaborators. Clinical course of untreated cerebral cavernous malformations: a meta-analysis of individual patient data. *Lancet Neurol* 2015 Published Online December 1, 2015 http://dx.doi.org/10.1016/S1474-4422(15)00303-8

Literature search strategies

OVID MEDLINE

1. Hemangioma, Cavernous, Central Nervous System/
2. Hemangioma, Cavernous/
3. (cavernous adj5 (angioma$ or hemangioma$ or malformation$)).tw.
4. cavernoma$.tw.
5. 2 or 3 or 4
6. exp brain/ or central nervous system/ or exp cerebral arteries/
7. exp brain neoplasms/
8. (brain$ or cerebral or intracerebral or central nervous system or intracranial or cerebellar or intraventricular or supratentorial).tw.
9. 6 or 7 or 8
10. 5 and 9
11. 1 or 10

EMBASE

1. Brain Hemangioma/
2. brain ventricle cavernoma/
3. cavernous hemangioma/
4. (cavernous adj5 (angioma$ or hemangioma$ or malformation$)).tw.
5. cavernoma$.tw.
6. 3 or 4 or 5
7. central nervous system/ or exp brain/ or exp brain ventricle/ or exp brain artery/
8. exp brain tumor/
9. (brain$ or cerebral or intracerebral or central nervous system or intracranial or cerebellar or intraventricular or supratentorial).tw.
10. 7 or 8 or 9
11. 6 and 10
12. 1 or 2 or 11

**Publications of cohorts eligible for inclusion in individual patient data meta-analysis**				
**Study**	**Date**	**Patients**	**Study design**	**Other selection criteria**
**Robinson**[WA18]	1991	66	Retrospective; hospital-based	None
**Zabramski**[WA24]	1994	21	Prospective; 6 families	Familial CCM
**Aiba**[WA21]	1995	110	Retrospective; hospital-based	None
**Kondziolka**[WA22]	1995	122	Retrospective + prospective; hospital-based	Conservative management
**Kim**[WA25]	1997	62	Retrospective; hospital-based	None
**Porter**[WA34*]	1997	110[†]	Retrospective + prospective; tertiary referral centre	None
**Moriarity**[WA26]	1999	68	Prospective; hospital-based	None
**Porter**[WA27]	1999	100	Retrospective; hospital-based	Brainstem CCM location
**Barker**[WA28]	2001	136	Retrospective; hospital-based	Bled at presentation
**Hasegawa**[WA29]	2002	82	Retrospective + prospective; radiosurgery unit	High-risk, bled at presentation; pre-treatment
**Mathiesen**[WA19]	2003	68	Retrospective + prospective; hospital-based	Brainstem CCM location
**Wang**[WA20]	2003	137	Retrospective; hospital-based	Brainstem CCM location
**Ghannane**[WA30]	2007	79	Retrospective; hospital-based	None
**Flemming**[WA35*]	2012	292[†]	Retrospective; tertiary referral centre	None
**Schneble**[WA36*]	2012	87[†]	Retrospective; tertiary referral centre	None
**Al-Shahi Salman**[WA37*]	2012	135[†]	Prospective; population-based	None
**Al-Holou**[WA31]	2012	<50	Retrospective; hospital-based	Children and young adults (≤25 years)
**Riant**[WA23]	2013	~50	Prospective; hospital-based	Familial CCM
**Kalani**[WA32]	2013	64	Retrospective; hospital-based	Pregnant women
**Kondziolka**[WA33]	2013	122	Prospective; hospital-based	Asymptomatic patients
**Jeon**[WA38*]	2014	326	Retrospective; hospital-based	None
**Li**[WA39*]	2014	331[†]	Prospective; hospital-based	Brainstem CCM location

* Included in the individual patient data meta-analysis.

[†] Size of the published cohort. Some people in these six cohorts did not meet the inclusion criteria for this meta-analysis and were excluded. Porter *et al.*[WA34] and Al-Shahi Salman *et al.*[WA37] provided unpublished data for this meta-analysis.

Characteristics of included cohorts

Characteristic*	Scotland 1999–2003 (n=135)		Scotland 2006–2010 (n=160)		Hôpital Lariboisière (n=81)		Mayo Clinic (n= 267)		Toronto Western Hospital (n=345)		Seoul National University Hospital (n=326)		Beijing Tiantan Hospital (n=306)	
Median age at diagnosis, years (IQR)	41	32–53	46	34–60	42	28–59	46	31–62	42	33–54	55	43–64	37	29–47
Female sex	80	59%	77	48%	47	58%	143	54%	194	56%	181	56%	145	47%
Mode of presentation														
Incidental	62	46%	67	42%	47	59%	98	37%	101	29%	70	21%	16	5%
Seizure	35	26%	52	33%	10	12%	76	28%	69	20%	95	29%	0	0%
ICH	17	13%	31	19%	10	12%	64	24%	116	34%	107	33%	231	76%
FND	21	15%	10	6%	14	17%	29	11%	59	17%	54	17%	59	19%
Multiple CCM	24	18%	29	18%	27	33%	49	18%	79	23%	49	15%	25	8%
Primary CCM location														
Lobar	90	67%	120	75%	–	–	156	58%	189	55%	193	59%	0	0%
Deep	9	7%	4	2%	–	–	34	13%	30	9%	52	16%	0	0%
Cerebellum	19	14%	11	7%	–	–	14	5%	24	7%	36	11%	0	0%
Brainstem	17	12%	25	16%	17	21%	63	24%	102	29%	45	14%	306	100%
CCM management														
Surgery or stereotactic radiosurgery	23	17%	11	7%	4	5%	73	27%	40	12%	79	24%	83	27%
Conservative management	112	83%	149	93%	77	95%	194	73%	305	88%	247	76%	223	73%
First outcome event in untreated follow-up														
ICH	7	5%	7	4%	4	5%	20	7%	24	7%	52	16%	90	29%
FND	14	10%	6	4%	–	–	–	–	32	9%	–	–	–	–
No outcome event in 5-year follow-up	115	85%	147	92%	77	95%	247	93%	290	84%	274	84%	216	71%
Median censored follow-up, years (IQR)	5·0	2·6–5·0	3·9	2·9–5·0	2·2	0·7–4·2	4·5	1·1–5·0	3·9	1·8–5·0	2·1	1·3–3·3	4·2	2·2–5·0

* Values are *n* (%) unless stated otherwise. ICH denotes intracranial haemorrhage. FND denotes non-haemorrhagic focal neurological deficit.

**Assessment of each of the seven cohorts against each of eight risks of bias***							
	**Scotland 1999–2003**	**Scotland 2006–2010**	**Hôpital Lariboisière**	**Mayo Clinic**	**Toronto Western Hospital**	**Seoul National University Hospital**	**Beijing Tiantan Hospital**
Was selection of exposed and non-exposed patients drawn from the same population? (Criterion: all were adults diagnosed with CCM, validated by MRI)	Definitely yes	Definitely yes	Definitely yes	Definitely yes	Definitely yes	Definitely yes	Definitely yes
Can we be confident in the assessment of exposure? (Criteria: MRI was used to establish diagnosis, brainstem location, and CCM multiplicity; mode of presentation was determined by brain imaging; sex and age were recorded)	Definitely yes	Definitely yes	Definitely yes	Definitely yes	Definitely yes	Definitely yes	Definitely yes
Can we be confident that outcome of interest was not present at start of study? (Criterion: ICH/FND was described at inception and ICH was described during follow-up)	Definitely yes	Definitely yes	Definitely yes	Definitely yes	Definitely yes	Definitely yes	Definitely yes
Did the study match exposed and unexposed for all variables that are associated with the outcome of interest or did the statistical analysis adjust for these prognostic variables? (Not applicable because the analysis did not assume any associations were known)	na	na	na	na	na	na	na
Can we be confident in the assessment of the presence or absence of prognostic factors? (Criteria: MRI was used to establish diagnosis, brainstem location, and CCM multiplicity; mode of presentation was determined by brain imaging; sex and age were recorded)	Definitely yes	Definitely yes	Definitely yes	Definitely yes	Definitely yes	Definitely yes	Definitely yes
Can we be confident in the assessment of outcome? (Criterion: ICH was defined as symptomatic and confirmed by brain imaging)	Definitely yes	Definitely yes	Definitely yes	Definitely yes	Definitely yes	Definitely yes	Definitely yes
Was the follow-up of cohorts adequate? (Criterion: median follow-up was >2 years)	Definitely yes	Definitely yes	Probably yes	Definitely yes	Definitely yes	Probably yes	Definitely yes
Were co-interventions similar between groups? (Not applicable because follow-up was censored at the time of any intervention)	na	na	na	na	na	na	na

* An 8-item tool published by the Cochrane Methods Bias group: http://bmg.cochrane.org/sites/bmg.cochrane.org/files/uploads/Tool%20to%20Assess%20Risk%20of%20Bias%20in%20Cohort%20Studies.pdf

na = not applicable to the present study.

Estimated risk of intracranial haemorrhage in five-year follow-up of each cohort, by mode of presentation

Study	Risk factor	Number of adults	Events	5-year estimated risk (95% confidence interval)	Unadjusted hazard ratio (95% confidence interval)
**Scotland, 1999–2003**	ICH or FND presentation	38	5	15·9% (3·0 to 28·8)	
	Other presentation	97	2	2·5% (0 to 5·9)	7·5 (1·4 to 38·5)
**Scotland, 2006–2010**	ICH or FND presentation	41	6	18·3% (4·8 to 31·8)	
	Other presentation	119	1	0·9% (0 to 2·6)	19·3 (2·3 to 160·4)
**Hôpital Lariboisière**	ICH or FND presentation	24	1	4·2% (0 to 12·2)	
	Other presentation	57	3	10·8% (0 to 22·6)	0·8 (0·1 to 7·5)
**Mayo Clinic**	ICH or FND presentation	93	15	20·7% (11·2 to 30·1)	
	Other presentation	174	5	4·3% (0·6 to 7·9)	6·4 (2·3 to 17·6)
**Toronto Western Hospital**	ICH or FND presentation	175	23	15·8% (9·7 to 21·9)	
	Other presentation	170	1	0·6% (0 to 1·8)	23·4 (3·2 to 173·1)
**Seoul National University Hospital**	ICH or FND presentation	161	42	33·5% (24·2 to 42·8)	
	Other presentation	165	10	10·2% (2·8 to 17·6)	5·0 (2·5 to 9·9)
**Beijing Tiantan Hospital**	ICH or FND presentation	290	88	32·9% (27·1 to 38·6)	
	Other presentation	16	2	14·4% (0 to 33·1)	3·0 (0·7 to 12·2)

Estimated risk of intracranial haemorrhage in five-year follow-up of each cohort, by primary CCM location

Study	Risk factor	Number of adults	Events	5-year estimated risk (95% confidence interval)	Unadjusted hazard ratio (95% confidence interval)
**Scotland, 1999–2003**	Brainstem	17	3	18·4% (0 to 37·3)	5·1 (1·1 to 22·6)
	Other location	118	4	4·2% (0·1 to 8·2)	
**Scotland, 2006–2010**	Brainstem	25	6	29·3% (8·9 to 49·7)	36·0 (4·3 to 299·1)
	Other location	135	1	0·8% (0 to 2·4)	
**Hôpital Lariboisière**	Brainstem	17	3	25·1% (0 to 50·8)	10·0 (1·0 to 96·8)
	Other location	64	1	3·1% (0 to 9·2)	
**Mayo Clinic**	Brainstem	63	10	19·5% (8·6 to 30·5)	3·7 (1·5 to 8·9)
	Other location	204	10	7·2% (2·9 to 11·5)	
**Toronto Western Hospital**	Brainstem	102	16	18·3% (10·0 to 26·6)	5·1 (2·2 to 11·9)
	Other location	243	8	4·3% (1·3 to 7·3)	
**Seoul National University Hospital**	Brainstem	45	11	29.5% (13.2 to 45.9)	1·8 (0·9 to 3·6)
	Other location	281	41	20.5% (14.0 to 27.1)	
**Beijing Tiantan Hospital**	Brainstem	306	90	31.9% (26.3 to 37.5)	–
	Other location	0	0	–	

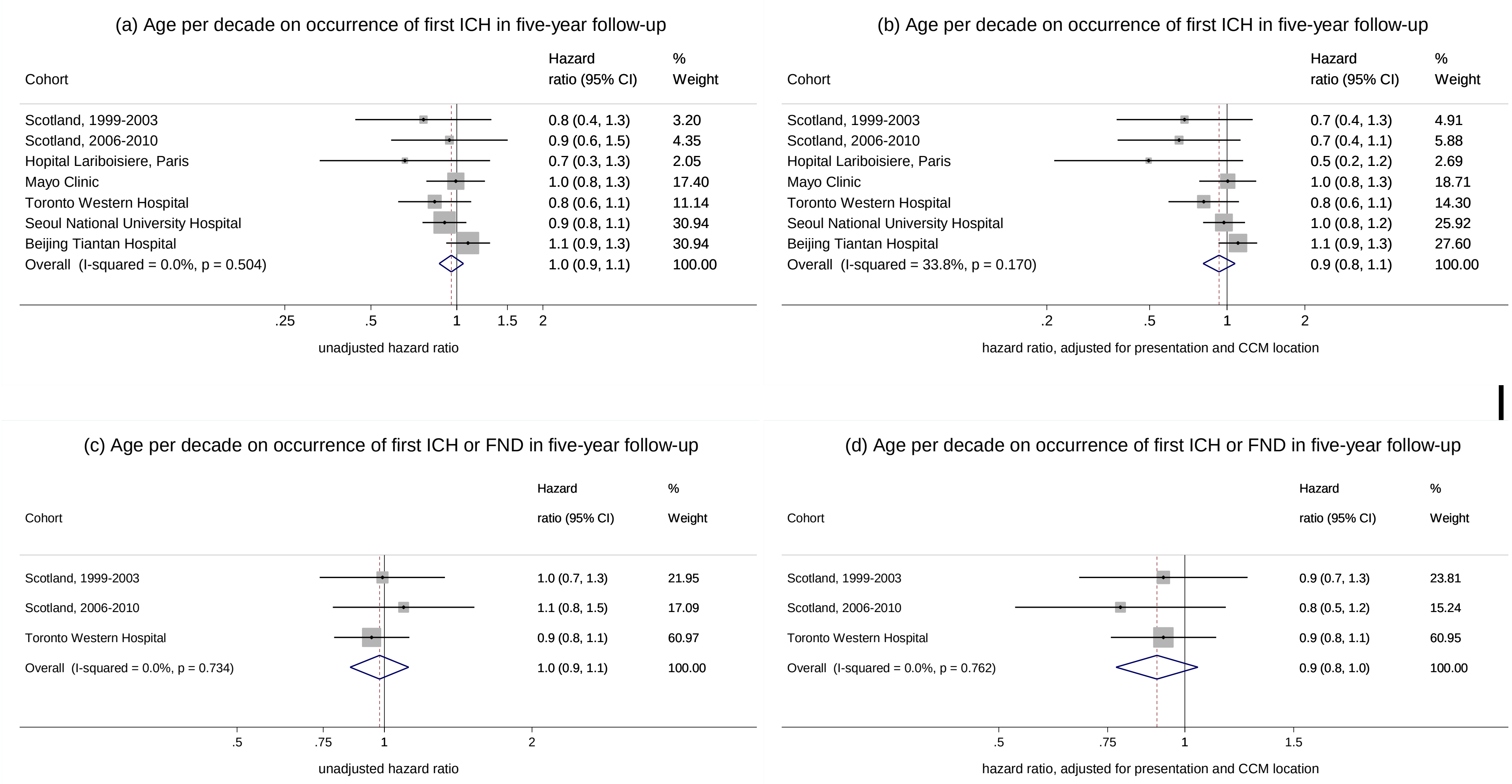

Forest plots displaying cohort-level and pooled estimates of associations between outcome over five years and age per decade, unadjusted (a and c) and adjusted for the two core predictors (b and d).

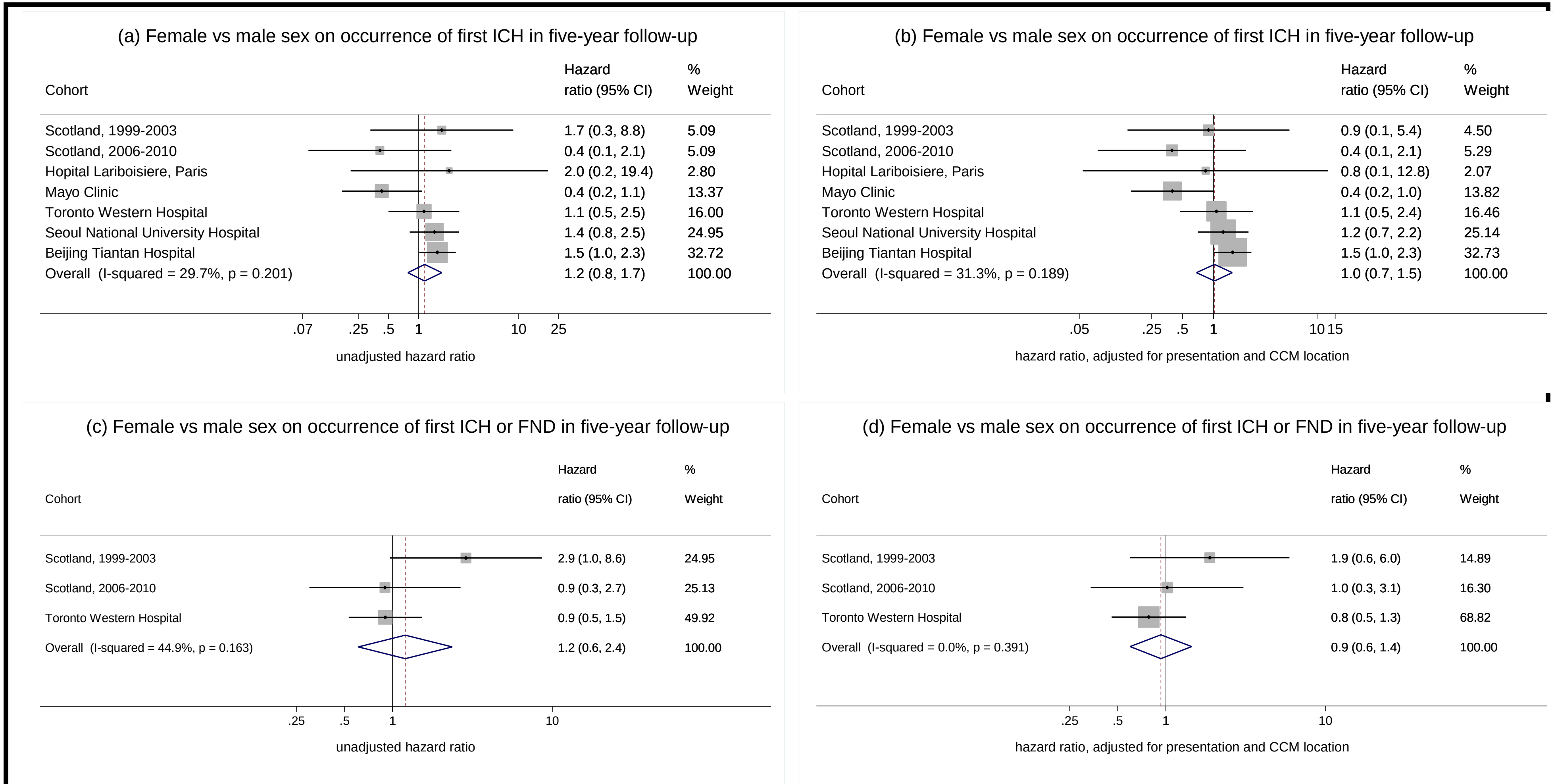

Forest plots displaying cohort-level and pooled estimates of associations between outcome over five years and sex, unadjusted (a and c) and adjusted for the two core predictors (b and d).

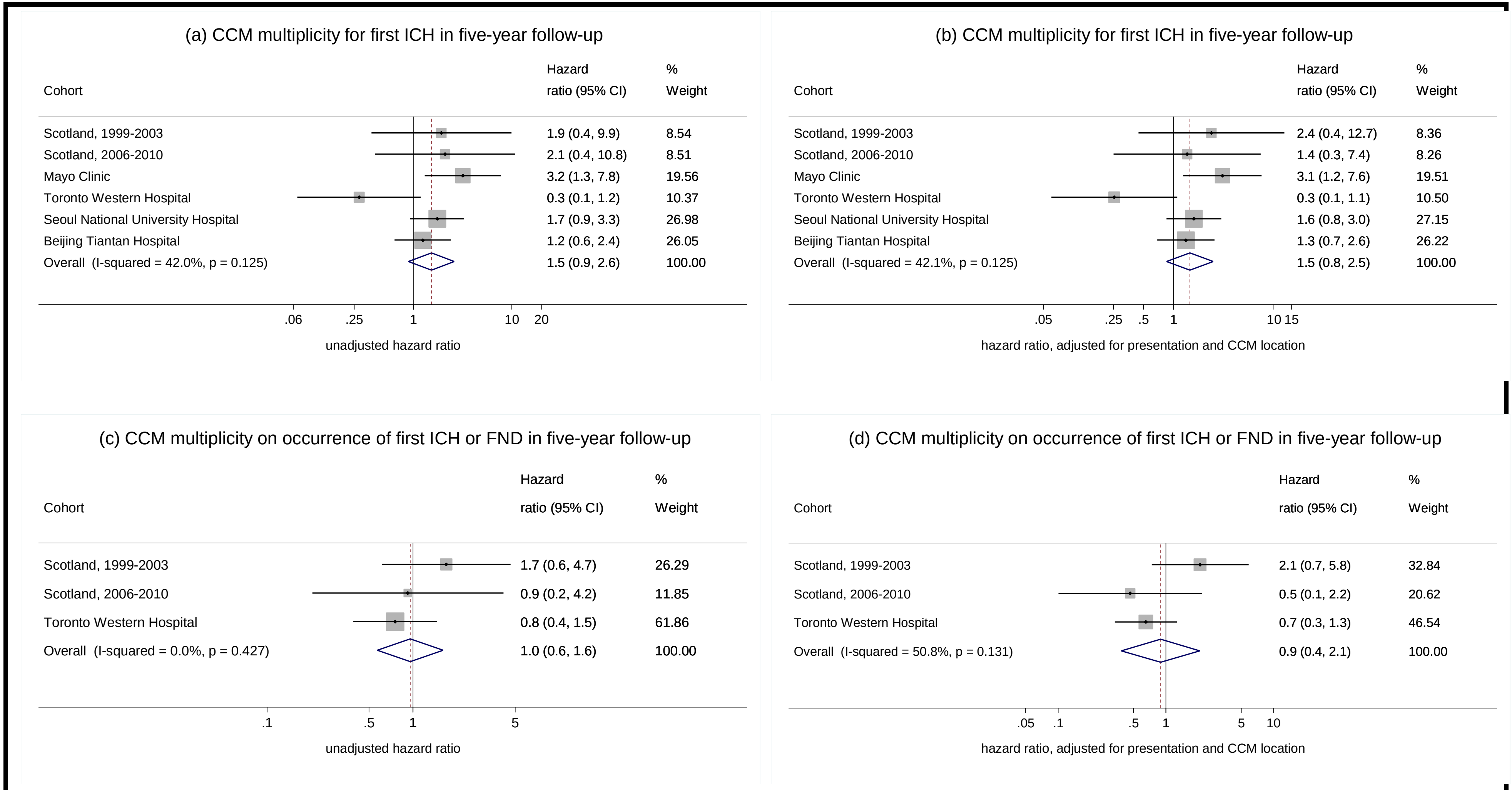

Forest plots displaying cohort-level and pooled estimates of associations between outcome over five years and CCM multiplicity, unadjusted (a and c) and adjusted for the two core predictors (b and d).

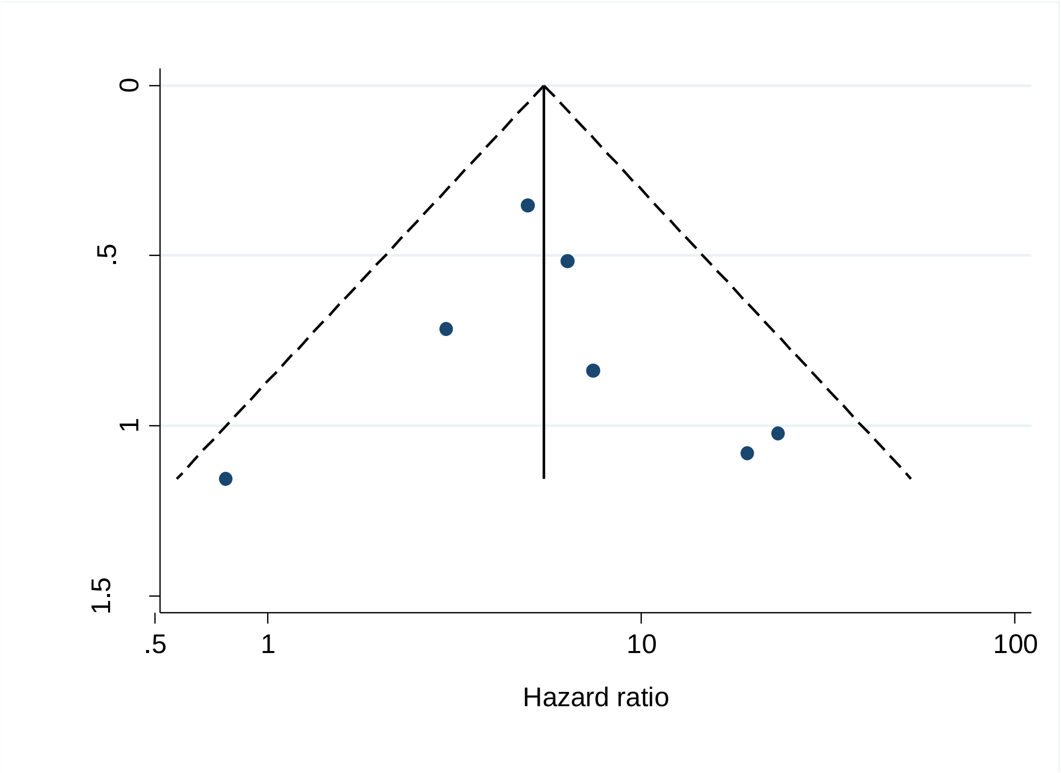

Funnel plot based on the association between the primary outcome over five years and intracranial haemorrhage or focal neurological deficit vs other presentation, in each of the seven included cohorts

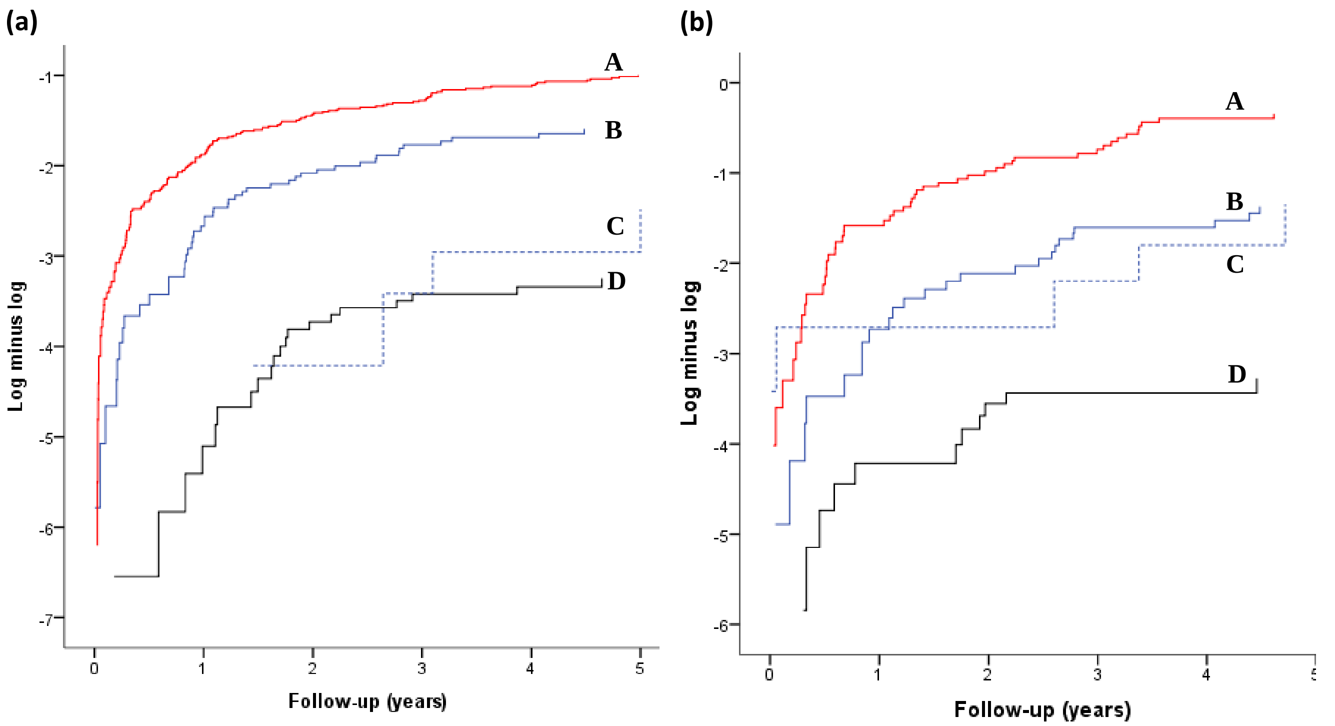

A – ICH or FND presentation, brainstem location; B – ICH or FND presentation, other location; C – Other presentation, brainstem location; D – Other presentation, other location

These two plots show the complementary logarithmic function of the survival function $S(t)$ – i.e. $\log\{-\log[S(t)]\}$ – (on the y-axis), plotted against the five-year follow-up (on the x-axis); $S(t)$ is the Kaplan-Meier estimate of the survival function at each successive occurrence of an ICH (a) or ICH or FND (b) at time t. These plots are used to test the assumption of proportional hazards before using Cox proportional hazards regression.

Tests of proportional hazards assumption using complementary log plots: (a) for an outcome of intracranial haemorrhage by presentation and location and (b) for an outcome of intracranial haemorrhage or focal neurological deficit by presentation and location

Estimated risk of intracranial haemorrhage, and intracranial haemorrhage or focal neurological deficit in five-year follow-up of each cohort, by core predictors

Cohort	Other presentation, other location		Other presentation, brainstem location		ICH or FND presentation, other location		ICH or FND presentation, brainstem location	
	Risk (%)	(95% CI)	Risk (%)	(95% CI)	Risk (%)	(95% CI)	Risk (%)	(95% CI)
**Outcome: ICH***								
**Scotland, 1999–2003**	2·7	0 to 6·3	—	—	10·0	0 to 23·5	26·7	0·7 to 52·6
**Scotland, 2006–2010**	1·0	0 to 2·9	—	—	—	—	46·9	17·5 to 76·3
**Hôpital Lariboisière, Paris**	3·8	0 to 11·2	46·7	0 to 95·3	—	—	11·1	0 to 31·6
**Mayo Clinic**	4·6	0·6 to 8·5	—	—	17·8	3·5 to 32·0	24·0	10·9 to 37·0
**Toronto Western Hospital**	0·7	0 to 2·0	—	—	10·3	2·9 to 17·7	21·7	12·1 to 31·3
**Seoul National University Hospital**	11·0	3·2 to 18·8	—	—	31·2	21·2 to 41·2	43·0	20·3 to 65·7
**Beijing Tiantan Hospital**	—	—	14·4	0 to 33·0	—	—	32·9	27·2 to 38·6
**Pooled cohorts**	3·8	2·1 to 5·5	8·0	0·1 to 15·9	18·4	13·3 to 23·5	30·8	26·3 to 35·2
**Outcome: ICH or FND**								
**Scotland, 1999–2003**	8·9	2·6 to 15·1	20·0	0 to 55·1	25·1	5·6 to 44·6	58·3	30·4 to 86·2
**Scotland, 2006–2010**	2·9	0 to 6·0	—	—	—	—	86·3	62·2 to 100
**Toronto Western Hospital**	0·7	0 to 2·0	42·8	5·6 to 79·9	27·3	16·3 to 38·2	44·4	32·1 to 56·7
**Pooled cohorts**	3·7	1·5 to 5·9	22·9	3·7 to 42·2	22·4	14·2 to 30·6	50·7	40·1 to 61·4

***** 1,620 adults contributed data on ICH outcomes. 640 adults contributed data on ICH or FND outcomes.
ICH denotes intracranial haemorrhage. FND denotes non-haemorrhagic focal neurological deficit.

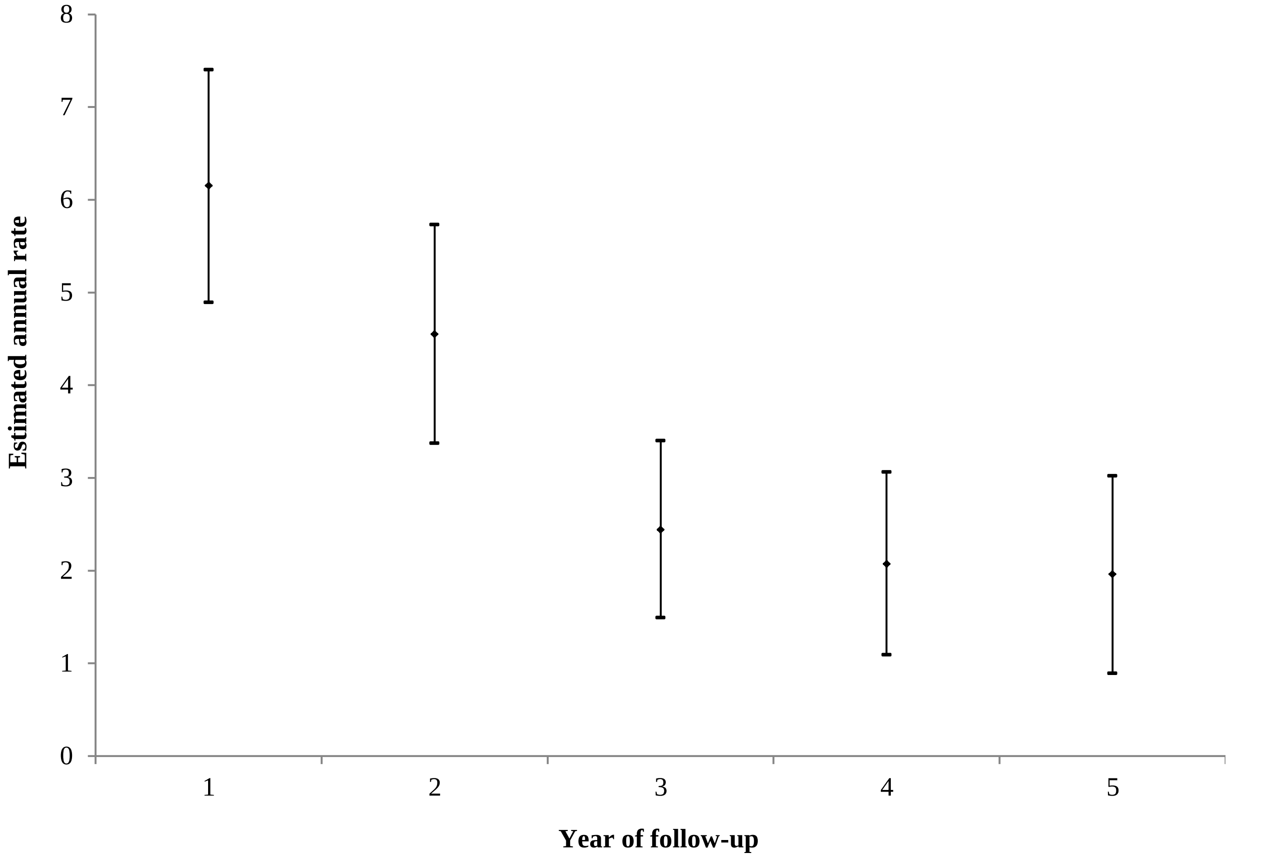

Estimates of the annual rate (95% confidence interval) of intracranial haemorrhage in each year of follow-up after initial CCM diagnosis for all 1,620 people in the entire dataset

**Estimates of the annual rate of experiencing an intracranial haemorrhage in each year of follow-up after initial CCM diagnosis by the two core predictors and for all 1,620 people in the entire dataset**					
**Risk factor**	**Year of follow-up**	**Number of adults at start of year**	**Number of outcome events in forthcoming year**	**Hazard rate (HR)* (%)**	**95% CI (HR) (%)**
**Other presentation, other location (n = 718)**	1st	718	4	0.59	0 to 1.17
	2nd	634	10	1.74	0.7 to 2.82
	3rd	516	4	0.85	0 to 1.69
	4th	422	1	0.27	0 to 0.79
	5th	331	1	0.32	0 to 0.96
**Other presentation, brainstem location (n = 80)**	1st	80	0	–	–
	2nd	74	1	1.48	0 to 4.39
	3rd	61	1	1.75	0 to 5.19
	4th	53	1	2.04	0 to 6.04
	5th	45	1	2.63	0 to 7.79
**ICH or FND presentation, other location (n = 327)**	1st	327	20	6.81	3.83 to 9.80
	2nd	260	13	5.57	2.54 to 8.59
	3rd	207	8	4.48	1.38 to 7.59
	4th	150	2	1.49	0 to 3.56
	5th	118	2	1.92	0 to 4.59
**ICH or FND presentation, brainstem location (n = 495)**	1st	495	68	15.09	11.52 to 18.67
	2nd	406	33	8.76	5.78 to 11.75
	3rd	347	12	3.77	1.64 to 5.91
	4th	289	13	4.98	2.27 to 7.69
	5th	233	9	4.20	1.46 to 6.94
**Entire dataset (n = 1,620)**	1st	1,620	92	6.15	4.89 to 7.40
	2nd	1,374	57	4.55	3.37 to 5.73
	3rd	1,131	25	2.44	1.49 to 3.40
	4th	914	17	2.07	1.09 to 3.06
	5th	727	13	1.96	0.89 to 3.02

*Hazard rate is an estimate of the risk of experiencing an ICH during year *x*, given that an individual has survived to the start of year *x*.

Estimated risk of intracranial haemorrhage or focal neurological deficit in five-year follow-up of each cohort, by mode of presentation						
**Study**	**Risk factor**	**Number of adults**	**Events**	**5-year estimated risk**	**95% confidence interval**	**Unadjusted hazard ratio (95% confidence interval)**
**Scotland, 1999–2003**	ICH/FND presentation	38	12	37·6%	20·5 to 54·7	4·7 (1·9 to 11·5)
	Other presentation	97	8	9·4%	3·2 to 15·6	
**Scotland, 2006–2010**	ICH/FND presentation	41	10	30·8%	14·5 to 47·2	11·5 (3·2 to 41·9)
	Other presentation	119	3	2·7%	0 to 5·6	
**Toronto Western Hospital**	ICH/FND presentation	175	50	35·6%	27·2 to 43·9	11·3 (4·5 to 28·3)
	Other presentation	170	5	4·5%	0·4 to 8·6	
**Pooled cohorts, un-stratified**	ICH/FND presentation	254	72	35·3%	28·4 to 42·1	8·2 (4·8 to 14·2)
	Other presentation	386	16	5·2%	2·6 to 7·8	

Estimated risk of intracranial haemorrhage or focal neurological deficit in five-year follow-up of each cohort, by primary CCM location						
**Study**	**Risk factor**	**Number of adults**	**Events**	**5-year estimated risk**	**95% confidence interval**	**Unadjusted hazard ratio (95% confidence interval)**
**Scotland, 1999–2003**	Brainstem	17	8	47·1%	23·3 to 70·8	5·2 (2·1 to 12·7)
	Other location	118	12	12·1%	5·7 to 18·6	
**Scotland, 2006–2010**	Brainstem	25	10	50·3%	26·7 to 73·9	22·6 (6·2 to 82·5)
	Other location	135	3	2·4%	0 to 5·1	
**Toronto Western Hospital**	Brainstem	102	35	43·4%	31·7 to 55·0	4·9 (2·9 to 8·6)
	Other location	243	20	10·7%	6·1 to 15·4	
**Pooled cohorts, unstratified**	Brainstem	144	53	44·5%	35·0 to 54·0	6·3 (4·1 to 9·6)
	Other location	496	35	8·8%	6·0 to 11·7	

Articles eligible for inclusion

A. Full text articles excluded

i. Data already included in a separate publication of an eligible cohort

WA1 Li D, Yang Y, Hao S-Y, et al. Hemorrhage risk, surgical management, and functional outcome of brainstem cavernous malformations. *J Neurosurg* 2013; **119**(4): 996–1008.

WA2 Li D, Hao S-Y, Tang J, et al. Clinical course of untreated pediatric brainstem cavernous malformations: hemorrhage risk and functional recovery. *J Neurosurg Pediatr* 2014; **13**(5): 471–83.

WA3 Moore SA, Brown RD, Jr., Christianson TJH, Flemming KD. Long-term natural history of incidentally discovered cavernous malformations in a single-center cohort. *J Neurosurg* 2014; **120**(5): 1188–92.

WA4 Fritschi JA, Reulen HJ, Spetzler RF, Zabramski JM. Cavernous Malformations of the Brain-Stem – a Review of 139 Cases. *Acta Neurochir (Wien)* 1994; **130**(1–4): 35–46.

ii. Diagnosis not certain

WA5 Abe M, Kjellberg RN, Adams RD. Clinical presentations of vascular malformations of the brain stem: comparison of angiographically positive and negative types. *J Neurol Neurosurg Psychiatry* 1989; **52**(2): 167.

WA6 Lobato R, Perez C, Rivas J, Cordobes F. Clinical, radiological, and pathological spectrum of angiographically occult intracranial vascular malformations. Analysis of 21 cases and review of the literature. *J Neurosurg* 1988; **68**(4): 518–31.

iii. Retrospective lifetime period at risk

WA7 Bruneau M, Bijlenga P, Reverdin A, et al. Early surgery for brainstem cavernomas. *Acta Neurochir (Wien)* 2006; **148**(4): 405–14.

WA8 Ferroli P, Sinisi M, Franzini A, Giombini S, Solero C, Broggi G. Brainstem cavernomas: Long-term results of microsurgical resection in 52 patients. *Neurosurgery* 2005; **56**(6): 1203–12.

WA9 Curling OD, Jr, Kelly DL, Elster AD, Craven TE. An Analysis of the Natural History of Cavernous Angiomas. *J Neurosurg* 1991; **75**: 702–8.

WA10 Murillo-Bonilla L, Cantu-Brito C, Arauz-Gongora A, Higuera-Calleja J, Padilla-Rubio J, Barinagarrementeria-Aldatz F. [Cavernous angioma. Clinical observations and prognosis of 133 patients]. *Rev Invest Clin* 2003; **55**(4): 387–93.

WA11 Cantu C, Murillo-Bonilla L, Arauz A, Higuera J, Padilla J, Barinagarrementeria F. Predictive factors for intracerebral hemorrhage in patients with cavernous angiomas. *Neurol Res* 2005; **27**(3): 314–18.

iv. Outcome events were not objective and symptomatic

WA12 Labauge P, Brunereau L, Laberge S, Houtteville JP. Prospective follow-up of 33 asymptomatic patients with familial cerebral cavernous malformations. *Neurology* 2001; **57**(10): 1825–28.

WA13 Kupersmith MJ, Kalish H, Epstein F, et al. Natural history of brainstem cavernous malformations. *Neurosurgery* 2001; **48**(1): 47–53.

WA14 Labauge P, Brunereau L, Levy C, Laberge S, Houtteville JP. The natural history of familial cerebral cavernomas: a retrospective MRI study of 40 patients. *Neuroradiology* 2000; **42**(5): 327–32.

v. Duration of follow-up was not adequately quantified

WA15 Feiz-Erfan I, Zabramski JM, Lanzino G, Porter RW. Natural history of cavernous malformations of the brain. *Operative Techniques in Neurosurgery* 2002; **5**(3): 171–75.

WA16 Ebrahimi A, Etemadifar M, Ardestani PM, Maghzi AH, Jaffe S, Nejadnik H. Cavernous angioma: a clinical study of 35 cases with review of the literature. *Neurol Res* 2009; **31**(8): 785-93.

WA17 Abdulrauf SI, Kaynar MY, Awad IA. A comparison of the clinical profile of cavernous malformations with and without associated venous malformations. *Neurosurgery* 1999; **44**(1): 41–46.

B. Cohorts eligible for inclusion invited to share but did not provide individual patient data

i. Agreed initially, but no response

WA18 Robinson JR, Awad IA, Little JR. Natural history of the cavernous angioma. *J Neurosurg* 1991; **75**: 709–14.

WA19 Mathiesen T, Edner G, Kihlström L. Deep and brainstem cavernomas: a consecutive 8-year series. *J Neurosurg* 2003; **99**(1): 31–37.

WA20 Wang C-C, Liu A, Zhang J-T, Sun B, Zhao Y-L. Surgical management of brain-stem cavernous malformations: report of 137 cases. *Surg Neurol* 2003; **59**(6): 444–54.

ii. Data no longer available

WA21 Aiba T, Tanaka R, Koike T, Kameyama S, Takeda N, Komata T. Natural history of intracranial cavernous malformations. *J Neurosurg* 1995; **83**(1): 56–59.

WA22 Kondziolka D, Lunsford LD, Kestle JRW. Natural history of cerebral cavernous malformations. *J Neurosurg* 1995; **83**(5): 820–24.

iii. Clinical data not available

WA23 Riant F, Bergametti F, Fournier HD, et al. CCM3 mutations are associated with early-onset cerebral hemorrhage and multiple meningiomas. *Mol Syndromol* 2013; **4**(4): 165–72.

iv. No response

WA24 Zabramski JM, Wascher TM, Spetzler RF, et al. The natural history of familial cavernous malformations: results of an ongoing study. *J Neurosurg* 1994; **80**(3): 422–32.

WA25 Kim D-S, Park Y-G, Choi J-U, Chung S-S, Lee K-C. An Analysis of the Natural History of Cavernous Malformations. *Surg Neurol* 1997; **48**: 9–18.

WA26 Moriarity JL, Wetzel M, Clatterbuck RE, et al. Natural history of cavernous malformations: a prospective study of 68 patients. *Neurosurgery* 1999; **44**(6): 1166–71.

WA27 Porter RW, Detwiler PW, Spetzler RF, et al. Cavernous malformations of the brainstem: experience with 100 patients. *J Neurosurg* 1999; **90**(1): 50–58.

WA28 Barker II FG, Amin-Hanjani S, Butler WE, et al. Temporal clustering of hemorrhages from untreated cavernous malformations of the central nervous system. *Neurosurgery* 2001; **49**: 15–25.

WA29 Hasegawa T, McInerney J, Kondziolka D, Lee JYK, Flickinger JC, Lunsford LD. Long-term Results after Stereotactic Radiosurgery for Patients with Cavernous Malformations. *Neurosurgery* 2002; **50**(6): 1190–98.

WA30 Ghannane H, Khalil T, Sakka L, Chazal J. Analyse d'une série de cavernomes du système nerveux central : 39 cas non opérés, 39 cas opérés et un cas décédé. *Neurochirurgie* 2007; **53**(2-3, Part 2): 217–22.

WA31 Al-Holou WN, O'Lynnger TM, Pandey AS, et al. Natural history and imaging prevalence of cavernous malformations in children and young adults. *J Neurosurg Pediatr* 2012; **9**(2): 198–205.

WA32 Kalani MYS, Zabramski JM. Risk for symptomatic hemorrhage of cerebral cavernous malformations during pregnancy. *J Neurosurg* 2013; **118**(1): 50–55.

WA33 Kondziolka D, Monaco EA, III, Lunsford LD. Cavernous malformations and hemorrhage risk. *Prog Neurol Surg* 2013; **27**: 141–46.

C. Cohorts providing individual patient data

WA34 Porter PJ, Willinsky RA, Harper W, Wallace MC. Cerebral cavernous malformations: natural history and prognosis after clinical deterioration with or without hemorrhage. *J Neurosurg* 1997; **87**(2): 190–97.

WA35 Flemming KD, Link MJ, Christianson TJH, Brown Jr RD. Prospective hemorrhage risk of intracerebral cavernous malformations. *Neurology* 2012; **78 (9)**: 632–36.

WA36 Schneble HM, Soumare A, Herve D, et al. Antithrombotic therapy and bleeding risk in a prospective cohort study of patients with cerebral cavernous malformations. *Stroke* 2012; **43 (12)**: 3196–99.

WA37 Al-Shahi Salman R, Hall JM, Horne MA, et al. Untreated clinical course of cerebral cavernous malformations: a prospective, population-based cohort study. *Lancet Neurol* 2012; **11**: 217–24.

WA38 Jeon J, Kim J, Chung Y, et al. A risk factor analysis of prospective symptomatic haemorrhage in adult patients with cerebral cavernous malformation. *J Neurol Neurosurg Psychiatry* 2014; **85**: 1366–70.

WA39 Li D, Hao S-Y, Jia G-J, Wu Z, Zhang L-W, Zhang J-T. Hemorrhage risks and functional outcomes of untreated brainstem cavernous malformations. *J Neurosurg* 2014; **121**(1): 32–41.
